# Applicability of the Cumberland Ankle Instability Tool in Elite Volleyball Athletes: A Cross-Sectional Observational Study

**DOI:** 10.3390/sports12030071

**Published:** 2024-03-05

**Authors:** Flavia Figlioli, Giacomo Belmonte, Valerio Giustino, Alberto Canzone, Elena Ferrantello, Marco Gervasi, Eneko Fernández-Peña, Giuseppe Battaglia, Antonino Bianco, Antonino Patti

**Affiliations:** 1 Sport and Exercise Sciences Research Unit, Department of Psychology, Educational Science and Human Movement, University of Palermo, 90144 Palermo, Italy; flavia.figlioli@unipa.it (F.F.); giacomobelmonte10@gmail.com (G.B.); alberto.canzone98@gmail.com (A.C.); elena.ferrantello@community.unipa.it (E.F.); giuseppe.battaglia@unipa.it (G.B.); antonino.bianco@unipa.it (A.B.); antonino.patti01@unipa.it (A.P.); 2 Department of Biomolecular Sciences—Division of Exercise and Health Sciences, University of Urbino Carlo Bo, 61029 Urbino, Italy; marco.gervasi@uniurb.it; 3 Department of Physical Education and Sport, University of the Basque Country UPV/EHU, 01007 Vitoria-Gasteiz, Spain; eneko.fernandezp@ehu.eus

**Keywords:** injury, CAIT, ankle instability, sport performance, volleyball

## Abstract

Background: The ease of administration of the Cumberland Ankle Instability Tool (CAIT) could represent a methodology for periodically evaluating athletes, preventing ankle instability injuries. This study aimed to achieve three objectives: (a) to evaluate the applicability of the CAIT scale in volleyball; (b) to explore whether ankle instability presents a greater risk in lower-level volleyball categories and whether elite athletes demonstrate an ability to mitigate this risk; and (c) to identify potential predictors of ankle instability. Methods: Eighty female volleyball players participated in this cross-sectional observational study. The CAIT was administered to evaluate the athletes belonging to some teams in Series A, B, and C. Results: The Spearman’s ranks correlation coefficient showed significant correlations between CAIT items. Additionally, the Cronbach’s α showed a high internal consistency. Our results showed a significant difference between athletes who reported pain and those who did not (*p* < 0.001). The multiple linear regression model analysis showed that pain was a predictor of ankle instability (*p* < 0.001). Conclusions: Our findings suggest that the CAIT can be used to evaluate ankle stability in volleyball players. This scale could represent a valuable tool for implementing specific intervention programs to prevent ankle injuries in athletes.

## 1. Introduction

Despite the common perception that ankle sprains are minor accidental events and quickly resolvable without long-term consequences, these injuries often lead to high rates of recurrence [1]. Sensorimotor deficits may persist for months or even years after the initial injury [2,3]. Recent studies highlight that the ankle is a body region subject to injuries in team sports such as volleyball, basketball, and soccer [4].

In volleyball, in fact, this joint is highly stressed, and traumas can accumulate, significantly affecting the athlete’s performance. Notably, a recent study found that the majority of injuries occur during blocking, often involving landing on an opponent. Landing-associated injuries primarily result from rapid inversion without plantar flexion [5]. Specifically in volleyball, the conflict zone surrounding the net is where the majority of ankle sprains occur after a vertical jump [6]. In a prospective study involving volleyball, basketball, and korfball players, Van den Doers et al. (2016) investigated the relationship between landing control and ankle sprain risk, finding that landing techniques with a greater ankle dorsiflexion moment and poor landing stability in the forward and diagonal jump direction are risk factors significant for ankle sprains [7].

Freeman (1965) classified chronic ankle instability into two main categories: functional instability and mechanical instability. Functional instability manifests with recurring symptoms such as a sense of giving way, weakness, pain, swelling, and difficulty walking on uneven surfaces. This condition results from compromised proprioception, perception, postural control, neuromuscular problems, and strength deficits. In contrast, mechanical instability results from problems within the musculoskeletal structures of the ankle, including ligament laxity resulting from previous sprains or partial/complete ligament tears [8]. Chronic ankle instability (CAI) occurs when, after one or multiple sprains, the ankle does not fully recover, leading to a decrease in the stability of the joint. As a result, recurrent events of ankle instability occur [9].

In volleyball, movements such as jumping and landing are fundamental [10]. Recovering ankle range of motion (ROM) accelerates rotation and increases muscle strength as well as proprioceptive and contraction capabilities. In fact, there is a correlation between the muscle strength of the ankle and the recovery of a healthy ROM of the ankle [11,12]. These repetitive movements subject the muscles and joints of the lower limbs to considerable stress. Consequently, volleyball players are subject to increased vulnerability to musculoskeletal injuries [13]. In 2021, Panoutsakopoulos et al. showed that having a flexible ankle joint can contribute to the performance of vertical squat jumps [14]. The authors suggested incorporating ankle flexibility training into the regimen of young volleyball players [14].

Training programs focus on strengthening the muscles surrounding the ankle since persistent ankle instability has a negative effect on the strength of the ankle muscles. However, as recent studies have demonstrated, strengthening the muscles surrounding the ankle becomes more challenging and the likelihood of the same injury recurring increases if ankle ROM and flexibility are not first restored [15].

Furthermore, Monteleone et al. (2023) showed that the cavus foot is associated with a higher risk of ankle sprain recurrence in female volleyball players [16]. The authors suggested that the cavus foot cannot adapt to changes in ground surfaces, making the ankle more susceptible to sprains [16].

Hiller et al. (2006) developed the Cumberland Ankle Instability Tool (CAIT), and demonstrated robust validity and reliability of the scale [17]. In detail, the CAIT showed excellent test–retest reliability (ICC_2,1_ = 0.96) and the construct validity and internal reliability were satisfactory (α = 0.83). Additionally, all items showed a point-measure correlation >0.5 and the item reliability index was 0.99 [17]. This tool was created specifically to evaluate both the left and right ankle, facilitating an assessment for each ankle [17]. An essential step in developing an effective prevention plan is to investigate the epidemiology of CAI. For this purpose, the International Ankle Consortium recommends three self-reported tools that are valid and reliable: The CAIT, The Ankle Instability Instrument (AII), and The Identification of Functional Ankle Instability (IdFAI) [18]. A measure with sufficient psychometric qualities can assist in a variety of tasks, including determining the extent of an injury, recovery time, whether an athlete is ready for competition, developing methods to prevent injuries, improving athlete’s well-being, and directing the rehabilitation process [19]. Gribble et al. (2014) suggested that, as a diagnostic tool, the CAIT has shown promise in differentiating patients with minor ankle sprains and those with CAI [20]. Various versions and adaptations of the CAIT are present in the literature [21,22,23,24,25,26,27,28,29,30]. However, to the best of our knowledge, this scale has rarely been administered in sports [31,32,33].

The objective of this cross-sectional observational study was threefold: (a) to evaluate the applicability of the CAIT scale in volleyball; (b) to examine differences in CAIT values among players of different volleyball categories; and (c) to identify potential predictors that can indicate ankle instability.

## 2. Materials and Methods

### 2.1. Study Design

This was a cross-sectional observational study. Volleyball players, ranging from 15 to 35 years old, were recruited from various teams and across different categories, including Series A, B, and C. Our study focuses exclusively on elite athletes, specifically those competing in Series A and B, the top Italian volleyball championships. However, it is important to note that although Serie C operates at a regional level, its participants possess a high level of skill and commitment. This distinction made it possible to analyze a sample characterized by players with different levels of technique, training, and performance. Specifically, we administered the CAIT to 80 athletes from the different categories.

**Figure 1:**
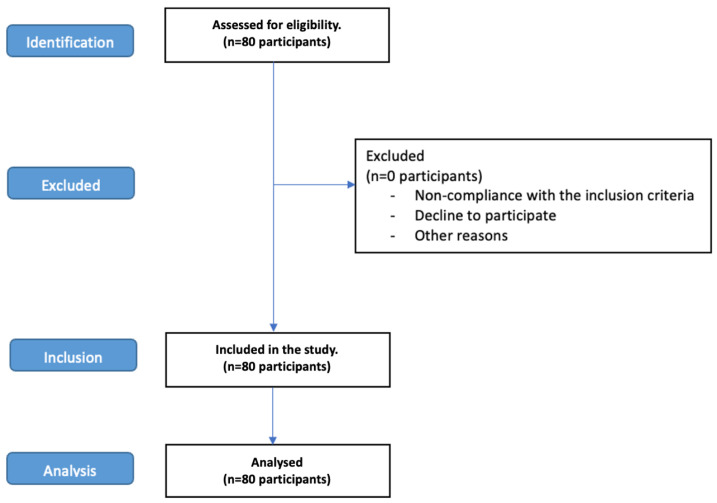
The STROBE flow chart.

The STROBE flow chart (Figure 1) was used to ensure that the assessment of participants of the study was conducted clearly [33].

The study was carried out in compliance with the principles of the Declaration of Helsinki and approved by the Bioethics Committee of the University of Palermo (n. 94/2022—Prot. 70310). Written informed consent was obtained from all participants before participating in the study.

### 2.2. Participants and Procedure

The STROBE guidelines were used to ensure a high-quality presentation of the conducted observational study (Figure 1) [34].

Eighty female volleyball players participated in this cross-sectional observational study (age (y): 25 ± 5.23; height (cm): 175 ± 0.08; weight (kg): 67.7 ± 9.73).

To be eligible for the study, participants had to meet the following inclusion criteria: (a) no bone fractures within the last 12 months [35]; and (b) playing regularly without injuries for at least three months. The participants were recruited voluntarily at the Sport and Exercise Sciences Research Centre of the University of Palermo. A researcher conducted interviews with participants to record general information, anthropometric measurements, details about previous injuries, and surgical interventions, to facilitate the administration of the CAIT scale. According to this information, the inclusion criteria were applied.

### 2.3. The Cumberland Ankle Instability Tool (CAIT)

The CAIT is a 9-item questionnaire designed to evaluate both ankles, i.e., pain in each ankle during daily activities, ankle instability in different types of physical activities, ankle control when recurring sprains occur, and the recovery period after recurrent ankle sprains. The nine items generate a total score from 0 to 30 for each foot, where 0 is the worst possible score, meaning severe instability, and 30 is the best possible score, meaning normal stability [25,36].

The Italian version of the CAIT was administered to the entire sample by a researcher who was unaware of the category in which each participant played [25]. The scales served to differentiate between stable and unstable ankles and assess the severity of functional instability encountered with a threshold of 27.5 points [17]. The questionnaire collected personal information such as the name, age, height, weight, sports participation, history of ankle sprains on both ankles, and the presence/absence of any associated painful symptoms. The CAIT investigates the perception of pain and ankle instability in relation to sports engagement, as well as the performance of daily activities such as going down stairs, running, walking, and overcoming uneven or flat surfaces.

The sample was stratified into two categories: (a) athletes with ankle injuries in the past and those without ankle injuries (Injury Group vs. No Injury Group); and (b) athletes who indicated the presence/absence of ankle pain (Pain Group vs. No Pain Group).

### 2.4. Statistical Analysis

All data were recorded in an Excel file (Microsoft Corporation, Redmond, WA, USA). Statistical analysis was performed with Jamovi software (version 2.3.21.0).

The distribution of quantitative data was assessed with the Shapiro–Wilk test (*p* > 0.05).

According to similar studies, the internal consistency of the CAIT was evaluated through Cronbach’s α [37,38]. For this assessment, the CAIT scores from all participants were utilized. An instrument demonstrates internal consistency when its items show at least moderate correlations with each other, and each item correlates with the total score (scores between 0.70 and 0.95 indicating good internal consistency) [39].

The Mann–Whitney U test was conducted to analyze the difference between participants reporting ankle injuries and those who did not, as well as between two groups categorized by the presence or absence of ankle pain. For each outcome, Cohen’s d was calculated to determine the effect size [40]. An effect size d = 0.2 corresponds to a small effect size; d = 0.5 medium; and d = 0.8 large [41]. The effect was calculated to further quantify the impact of pain or prior injuries on CAIT scores.

The relationship between the CAIT scores, previous ankle injuries, ankle pain, and volleyball series was analyzed using linear regressions, including each of these parameters as independent variables and the CAIT scores as dependent variables (regression *p*-values and analyzed R-values were calculated). The R^2^, or the coefficient of determination, is an index that measures the link between the variability of the data and the correctness of the statistical model used [42]. Typically, it ranges from 0 to 1, and a value of 1 means that all the variances in the dependent variable are explained by the independent variables, indicating a perfect fit of the model to the data [42].

## 3. Results

The post hoc sample size power analysis (α = 0.05 and f = 0.15) showed that, with a total sample size of 80 participants, we achieved a power of 82% [21].

Shapiro–Wilk’s normality test showed a non-Gaussian distribution of the CAIT values (*p* < 0.05).

**Figure 2:**
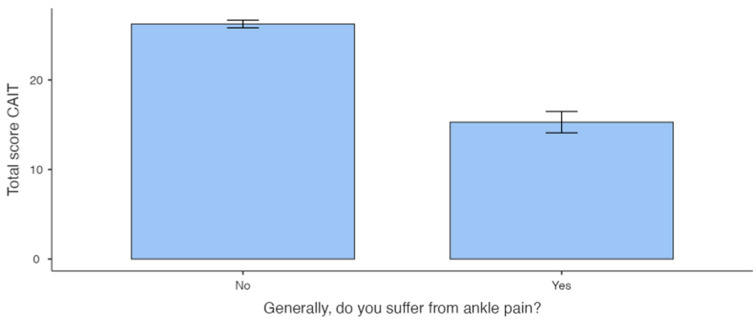
Analysis between the Pain Group and No Pain Group of right ankles.

**Figure 3:**
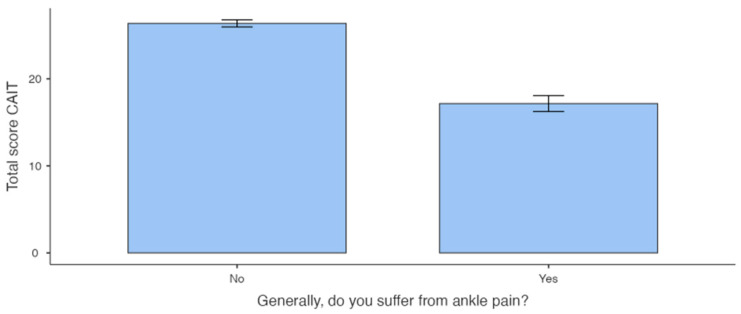
Analysis between the Pain Group and No Pain Group of left ankles.

The Mann–Whitney U analysis showed no significant differences between participants who reported ankle injury (Injury Group) and those who did not (No Injury Group). The same analysis showed a significant difference between participants who reported pain (Pain Group) and those who did not (No Pain Group) (Table 1A,B and Figure 2 and Figure 3).

The CAIT scores showed a Cronbach’s α value of 0.81 in the evaluations of the right ankle and 0.73 on those of the left ankle.

Multiple linear regression models for the right ankle are shown in Table 2. The adjusted R^2^ value of the regression, which included any previous ankle injuries, perception of pain, and series as independent variables and total CAIT scores as dependent variables, was 0.57 (Table 2). Similarly, multiple linear regression models for the left ankle are shown in Table 3 with R^2^ = 0.55.

## 4. Discussion

To the best of our knowledge, this is the first study that has applied the CAIT scale to evaluate ankle stability in volleyball players. This study primarily aimed to assess the effectiveness of the use of the CAIT scale in volleyball.

The Cronbach’s α values were used to assess internal consistency reliability [24,29]. In line with the literature, this suggests robust reliability on athletes’ responses and the functional evaluation of their ankles [29]. Cronbach’s α showed a value of 0.81 for the right ankle, which is a high internal consistency. The analysis for the left ankle showed a similar trend, with Cronbach’s α = 0.74. The internal consistency values of the CAIT obtained in this study are in line with those found in previous studies assessing the validity of the scale in other samples [21,22,23,24,25,26]. The evaluations of both ankles revealed consistent patterns, suggesting a potential interdependence where the instability of one ankle may contribute to the destabilization of the other.

Analysis comparing the Injury Group and the No Injury Group revealed no significant difference, suggesting that an injury event does not necessarily lead to ankle instability [43]. Conversely, when comparing athletes who reported chronic pain in ankles with those who did not, a notable difference in CAIT scores was detected. The group experiencing pain exhibited lower scores compared with the group without pain. In contrast to injuries, our data suggest that the presence of pain is indicative of a less stable ankle.

However, these conclusions are countertrended with some data present in the literature that indicate ankle instability as a common consequence of ankle sprain [23,32]. Other studies suggest more complex hypotheses about the cause of ankle instability [43]. To bolster this hypothesis, we conducted a comprehensive analysis of the variables by integrating the average score of the CAIT index as an independent variable. The analysis showed that pain can be a predictor of ankle instability. Nevertheless, injuries also exhibited moderate significance concerning CAIT scores of the right ankle. Research using the English CAIT scale suggested a cutoff score of 25.5 [36]. A cutoff score of 25.5 was also indicated in the Japanese CAIT scale [33]. In 2014, Wright and colleagues recalibrated the CAIT cutoff values, suggesting that a score ≤ 25 can effectively identify the presence/absence of chronic ankle instability [36]. On the other hand, in 2023, Huit et al. proposed a lower cutoff point to indicate possible ankle instability [24]. The authors indicated a value of 20.5 to differentiate healthy subjects from those with chronic ankle instability. The threshold scores for the Arabic CAIT and the Greek CAIT scales were 23.5 and 24.5, respectively. However, it should be noted that some scales, such as the Dutch CAIT, indicated a lower cutoff score of 11.5. Our results are in line with the observed trend. The group experiencing chronic pain exhibited a score of 15.3 for the right ankle and 17.2 for the left ankle. Similarly, participants who reported ankle injuries recorded a score of 17.2 for the right ankle and 20.2 for the left ankle. Our data indicate that individuals with a history of injury score better than those experiencing chronic pain.

The multiple linear regression analysis did not reveal any significant influence of the category, and consequently, the level of players on the risk of injuries. Thus, it appears that being part of a team of higher series does not necessarily guarantee a focus on training protocols for preventing ankle instability. However, we are aware that this is only a hypothesis to be confirmed, and the design of this study alone is insufficient to draw definitive conclusions. On the other hand, our conclusions are in line with those shown by Lin et al. (2022) [27]. The authors examined basketball players and found that the prevalence of chronic ankle instability remains consistent across different competitive levels and playing positions. This is attributed to the nature of basketball itself, which contributes to the high incidence of chronic ankle instability [27].

Another aspect is that the average ratings of the analyzed players did not pass the threshold values typically found in the literature for the CAIT scale [36]. These findings suggest a substantial risk of ankle instability. However, the variability in cutoff scores across different countries should be noted. Additionally, the study populations encompassed a diverse range of individuals, from the general population to athletes. This suggests that multiple cutoff criteria may be necessary to accurately discern between a stable and an unstable ankle across various study populations.

### Limitations

These findings warrant confirmation with a larger sample size. Moreover, it is imperative to delve deeper into the relationship between pain and injuries, conducting a thorough and detailed study for a more comprehensive evaluation. This is suggested by analysis of the multiple linear regression model, which moderately explains the relationship between the predictors. The R^2^ value of 0.57 in the right ankle and the R^2^ value of 0.55 in the left ankle indicate that only 57% and 55% of the observed variabilities in the target variable are accounted for by the regression model, respectively [42].

## 5. Conclusions

In conclusion, our findings suggest that the CAIT scale can be used to evaluate ankle stability in volleyball players. The ease of administration of the scale makes it a useful methodology for regularly assessing athletes. Consequently, it could represent a valuable tool for implementing specific intervention programs to prevent ankle injuries in athletes. In conclusion, the CAIT is easy to use and could be a useful tool for coaches to evaluate athlete’s ankle stability and prevent injuries.

## Figures and Tables

**Table 1:** Mann–Whitney U analysis between the Injury Group vs. No Injury Group and Pain Group vs. No Pain Group for both the right and left ankle.

A	Participants (n)	No Injury Group Total Score CAIT	Participants (n)	Injury Group Total Score CAIT	*p*	Effect Size
Right Ankle	66	22.3 ± 6.59	14	17.2 ± 9.99	0.084	0.295
Left Ankle	67	22.2 ± 5.82	13	20.2 ± 8.74	0.724	0.0631
B	Participants (n)	Pain Group Total Score CAIT	Participants (n)	No Pain Group Total Score CAIT	*p*	Effect Size
Right Ankle	35	15.3 ± 7.05	45	26.2 ± 2.88	<0.001	0.911
Left Ankle	39	17.2 ± 5.68	41	26.4 ± 2.61	<0.001	0.906

**Table 2:** Multiple linear regression model for the right ankle.

Predictor	Estimate	SE	t	*p*
Intercept	269.164	1.10	244.421	<0.001
Any previous ankle injuries?				
Yes–No	−33.289	1.52	−21.969	0.031
Generally, do you suffer from ankle pain?				
Yes–No	−106.563	1.15	−93.000	<0.001
Series:				
B–C	0.0681	1.31	0.0521	0.959
A–C	−10.445	1.48	−0.7060	0.482

**Table 3:** Multiple linear regression model for the left ankle.

Predictor	Estimate	SE	t	*p*
Intercept	26.397	1.167	22.624	<0.001
Any previous ankle injuries?				
Yes–No	−2.299	1.332	−1.726	0.089
Generally, do you suffer from ankle pain?				
Yes–No	−9.235	0.981	−9.416	<0.001
Series:				
B–C	0.497	1.281	0.388	0.699
A–C	0.429	1.286	−0.334	0.740

## Data Availability

The data presented in this study are available on request from the corresponding author.
